# Impact of particle size of multivesicular liposomes on the embolic and therapeutic effects in rabbit VX2 liver tumor

**DOI:** 10.1080/10717544.2022.2157519

**Published:** 2023-01-16

**Authors:** Hailing Tang, Changhui Cao, Guangyuan Zhang, Zhengkao Sun

**Affiliations:** a School of Pharmacy, Shanghai Jiao Tong University, Shanghai, China; b Department of Radiology, Fudan University Cancer Center, Department of Oncology, Shanghai Medical College, Fudan University, Shanghai, P.R. China; c Department of Orthopaedics, Qilu Hospital (Qingdao), Cheeloo College of Medicine, ShangDong University, Qingdao, China

**Keywords:** Embolic agent, multivesicular liposome, TACE, rabbit VX2 liver tumor, metronome chemotherapy

## Abstract

Transcatheter arterial chemoembolization (TACE) is usually considered more efficacious in the local treatment of parenchyma-sparing hepatocellular carcinoma (HCC). At present, embolic agents commonly used in TACE, include DC pellets, Hepasphere, Lipiodol, etc. Except that iodine oil is a viscous fluid embolic agent, other solid microsphere particles used clinically range from 70 to 700 µm, among which 100 to 300 µm is the most commonly used. With the technology development of micro-invasive interventional therapy, the specific distal embolization through TACE to occlude tumor arterial blood supply in patients with HCC is also required more accurately. Effective terminal embolization is considered to be a preferred option for TACE therapy due to significantly improving the survival rate of patients and preserving liver function. In this article, we prepared the multifunctional multivesicular liposomes (IVO-DOX-MVLs) (<100 µm) that can simultaneously encapsulate ioversol and doxorubicin based on the high-phase transition temperature (*T*_m_) lipid ingredients, and evaluated its local artery embolization and therapeutic effect in rabbit VX-2 tumor model. The influence of particle size on occlusion and therapeutic effect of MVLs on rabbit VX-2 liver tumor models were well evaluated, including the tumor volume change, tumor growth rate, and necrosis rate, which were evaluated by magnetic resonance (MR). MVL samples with average particle size distribution of 50–60 µm exhibited fewer off-target embolization. Through TACE, IVO-DOX-MVLs were directly transported to the tumor tissues, playing roles of embolization performance, CT imaging effect, and local tumor killing effect. The feasibility of MVLs as a multifunctional embolic agent in its clinical application can be further improved by optimization of lipid composition and preparation process.

## 1. Introduction

Hepatocellular carcinoma (HCC) is a highly prevalent malignant disease with high mortality rate, and is currently one of the top three cancers in the world in terms of mortality (Hucke et al., 2022). When diagnosed, only a small percentage of patients can be treated by surgical resection or liver transplantation to obtain a relatively ideal therapeutic effect (Tsai et al., 2019). The majority of patients with intermediate or advanced-stage HCC diseases are only eligible for parenchyma-sparing local therapies because of inadequate hepatic reserve or other medical comorbidities, including transcatheter arterial chemoembolization (TACE) (Bargellini et al., 2021), transcatheter arterial embolization (Hidaka et al., 2021), radiofrequency ablation (RFA) (Lu et al., 2022), ethanol injection (Takata et al., 2022), and transcatheter arterial infusion chemoembolization (TACBE) (Imamura et al., 2021). Although there have not been sufficient clinical data demonstrating the superiority of one therapy over the others, TACE is usually considered more efficacious by combining the effects of chemotherapy with embolization (Fan et al., 2021). Theoretically, TACE regiments should be designed to achieve specific artery vascular occlusion, and local delivery of chemotherapeutic drug into the tumor. However, the embolic agents currently used in clinic, whether microspheres or lipiodol, are directly mixed with drugs or contrast agents by simple adsorption and administered during the time of surgery. In this way, the loaded drug or contrast agent can be eluted quickly, which can realize the local high-concentration chemotherapy drug infusion during the embolization process. Different embolic agents have different systemic circulation characteristics of free drugs after TACE (Bargellini et al., 2021). Drugs mixed with Lipiodol, such as doxorubicin, will have significant drug loss into the systemic circulation, thus causing unwanted side effects and systemic toxicity. The combination of TACE with a variety of minimally invasive interventional therapies has been shown to significantly improve the overall survival of patients with HCC in clinical trials (Liu et al., 2015). Novel multifunctional embolic agents have been designed and demonstrated good local targeted therapeutic effects in preclinical studies. Chen et al. (2022) developed gelatin microspheres encapsulated Fe_3_O_4_ nanoparticles and doxorubicin, which not only has the effect of arterial embolization, but also can achieve rapid local drug release of doxorubicin in tumor tissues by heating Fe_3_O_4_ nanoparticles with microwave. Fe_3_O_4_ nanoparticles in gelatin microspheres also have enhanced MRI imaging function. In TACE-induced hypoxic tumor environment, ADM/Fe_3_O_4_-MS mainly induced ferroptosis of tumor cells. Shi et al. (2021) established rabbit VX-2 liver tumor model and compared the tumor inhibitory effects of lipiodol containing donafenib and epirubicin on tumor angiogenesis when treated with TACE. The results showed that the donafenib lipiodol group had a lower rate of tumor metastasis, a higher rate of tumor necrosis, a significantly reduced quantity of CD31-labeled tumor blood vessels, and promoted the infiltration of CD8^+^ T cells in the tumor area. This also provides a reference for the immune-based therapeutic mechanism of TACE.

Recently studies were indicating that drug eluting beads (DEB) with sustained drug release properties could be useful in TACE treatments. Comparing with lipiodol-based regimens, DEB (DC bead, HepaSphere, Tandem, Lifespearl) can minimize the amount of drugs that reach the systemic circulation, and significantly improve antitumoral efficacy (Odisio et al., 2015). DC beads in market were about 70–700 µm in size. Smaller size beads (<100 µm) were shown to have lower drug loading capacity, faster drug release, better terminal embolization effect, better permeability in tumor tissue, and higher concentration drug delivery in the vascular network of tumor tissue (Chang et al., 2021). To embolize tumor(s) as selectively as possible, smaller beads in the range of 50–100 µm would be more preferable. However, there are not enough clinical data to support whether it can significantly improve the overall survival rate of patients. Few reports mentioned the possible increased risk of hepatobiliary injury, such as bile duct dilatation, portal vein stenosis, and liver failure on the clinical side effects and complications (Chang et al., 2021). However, the overall safety and efficacy of small-caliber DEB in clinical use has been recognized by radiologist. Some researchers also reviewed the clinical efficacy of starch microspheres and polyvinyl alcohol microspheres less than 100 µm as TACE embolic agents. Using 50 ± 7 µm degradable starch microspheres instead of 300–500 µm drug-eluting beads (DEB), loading with the antitumor drug doxorubicin, had better overall response rate in the initial treatment, and could significantly reduce the serum AFP level. Lee et al. (2012) used a cocktail regimen of mixing three antitumor drugs (100 mg cisplatin, 50 mg doxorubicin, 10 mg mitomycin) with lipiodol and different PVA particles to inject directly into the tumor feeding arteries through TACE. The distribution characteristics of PVA microspheres with the size of 47–90 µm and 90–250 µm were observed by histopathological sections. The results of clinical studies showed that the small PVA microspheres deposited more in the end of the arterial capillaries of the tumor, and rarely leaked to the hepatic sinusoids.

**Figure 1.**
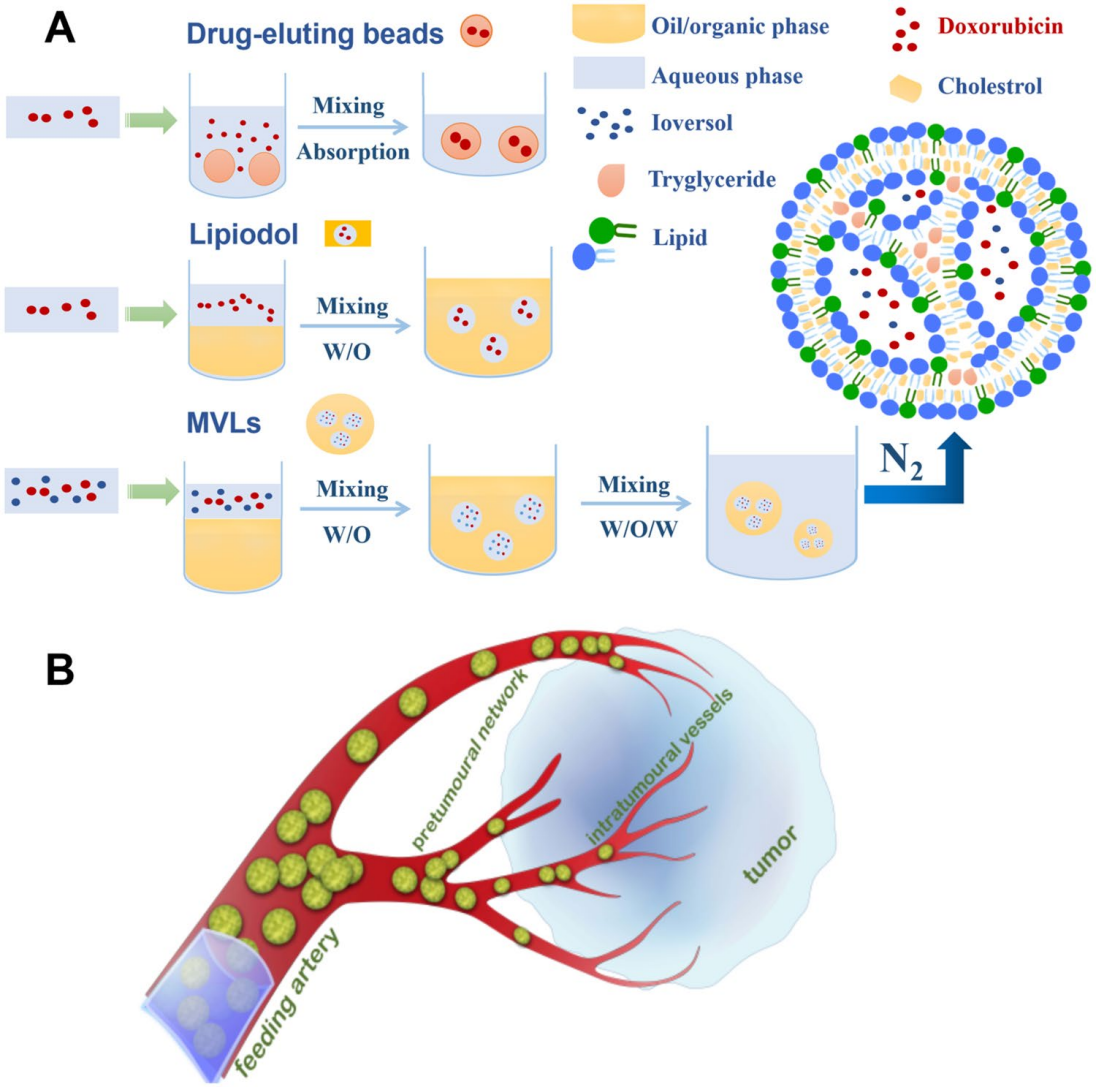
(A) Preparation of DC-beads and Lipiodol embolic agents currently in clinic vs. MVLs; (B) Schematic illustration of MVLs causing vascular occlusion. MVLs are released in the feeding artery and carried to intra-tumoral vessels by the blood flow. For its spherical shape, stable structure and good fluidity, MVLs make control of the occlusion site more accurate, allowing greater distal embolization after free-flow injection when compared with irregular, non-spherical embolic agents.

So based on these observations (Figure 1(A)), we hypothesized that it would be more desirable to prepare smaller drug-loaded microspheres with sustained drug release properties and can target more selective to smaller vessels feeding the tumor. We hope such a formulation would allow sustained release of chemotherapeutic drugs in targeting tumor tissues with minimal systemic exposure combined with calibrated tumor vessel obstruction.

We designed in this study a novel multivesicular liposomes (MVLs) embolization agent simultaneously encapsulating ioversol and doxorubicin. To achieve the embolic effect, the lipid formulations were adjusted to make the membrane more rigid and stable. Two different sizes of MVL preparations were made to investigate the embolic effect. Ioversol was loaded in MVL as a water-soluble contrast agent to present the precise position of MVLs in blood vessels under digital subtraction angiography (DSA). Cytotoxic drug doxorubicin was loaded together at low concentration but persistently for antiangiogenesis effect (Park et al., 2021). We evaluated the release profile of IVO-DOX-MVLs in vitro as well as their embolic effect when delivered intra-arterially into the VX2 liver tumor model in rabbits.

## 2. Materials and methods

### 2.1. Materials

Hydrogenated soybean phosphatidylcholine (HSPC) was obtained from Japan NOF Corporation (Tokyo, Japan). Cholesterol and 1,2-dipalmitoyl-*sn*-3-phospho glycerol (DPPG) were from Avanti Polar Lipids (AL, USA). Triolein was purchased from Shanghai Chemical Reagent Co. (Shanghai, China). l-Lysine was purchased from Sigma Aldrich Co. (USA). Ioversol injection (320 mg/mL) and ketamine (2 mL:0.1g) were from Jiangsu Hengrui Medicine Co., Ltd. (Lianyungang, China). Doxorubicin hydrochloride was from Shenzhen Wanle Pharmaceutical Co., Ltd (Shenzhen, China) and diazepam was from Xudonghaipu Medicine Co., Ltd. (Shanghai, China). All other reagents were of chemical pure or analytical grade. All the materials and reagents were used as received without any further purification.

### 2.2. Rabbit VX2 liver tumor

All the experimental procedures were approved by the Animal Care and Use Subcommittee at Fudan University and performed according to Institutional Guidelines. VX2 tumor strain was obtained as a gift from Liver Cancer Research Department of Zhongshan Hospital (Shanghai, China). Eighteen adult New Zealand white rabbits, male or female, weighing 1.8–2.3 kg, were provided by the Animal Research Center of the Medical School of Fudan University (Shanghai, China), including three carrier rabbits.

All surgical procedures and imaging examinations were conducted when the animals were intramuscularly anesthetized with a mixture of ketamine hydrochloride (0.1 g/kg) and diazepam (5 mg/kg). To implant VX2 tumor into the liver, the tumor was first grown in the hind leg of carrier rabbits for 3 weeks, then surgically excised from the carrier rabbit and placed in 0.9% sodium chloride. Tumor tissue was minced into approximately 1–2 mm^3^ fragments and suspended in 5 mL normal saline. A midline laparotomy was performed to expose the liver of the recipient rabbit, and tumor fragment was implanted in the left lobe of liver. The abdomen was closed in two layers. The tumors were allowed to grow to 1–2 cm in diameter which typically required 2 weeks. Fifteen rabbits with VX2 liver tumor were divided into three groups randomly. Group A was injected with small size MVLs suspension, Group B with large size MVLs suspension, and Group C with 0.9% sodium chloride at the dose of 0.5 mL/kg as a control group.

### 2.3. Preparation of MVLs containing ioversol and doxorubicin hydrochloride

A double-emulsion procedure (Li et al., 2019) was used to prepare MVLs containing ioversol and doxorubicin hydrochloride. Briefly, 1 mL of chloroform containing the lipids (41 mg HSPC:40.5 mg cholesterol:5 mg DPPG: 11.25 mg triolein) in 1 mL aqueous solution (the first aqueous solution) was emulsified by Scientz-IID Ultrasonic Homogenizer (Ningbo Scientz Biotechnology Co., Ltd. Ningbo, China) for 30 sec (30% power output, 25 °C) to produce a w/o emulsion. The first aqueous solution contains 1 mg of doxorubicin hydrochloride in 1 mL of 320 mg ioversol injection. This w/o emulsion (2 mL) was subsequently emulsified with 6 mL of the second aqueous solution containing 4% glucose (wt/vol) and 20 mM lysine at 2800 r/min by XHF-D mixer (Ningbo Scientz Biotechnology Co., Ltd. Ningbo, China) at 40 °C to prepare w/o/w emulsion. Then the w/o/w emulsion was diluted with 4 mL of second aqueous solution poured into 100-mL egg type flask. Chloroform was removed by flushing nitrogen over the surface of the double emulsion at 35–37 °C. The resultant MVLs were collected at 100*g* for 10 min, and resuspended in sterile saline solution after discarding the supernatant. BD Falcon™ cell strainers (BD Biosciences, USA) were used to isolate different size MVLs, improve the uniformity of MVLs. The ioversol and doxorubicin hydrochloride concentration in MVLs were determined by HPLC.

### 2.4. Particle characterization of MVLs

The MVLs suspensions were diluted in saline. Particle size distribution was measured by Mastersizer 2000 particle size analyzer (Malvern, Worcestershire, UK). The morphology was estimated by a Digital Biological Microscope (IX71-32PH Digital Biological Microscope, Olympus Co. Ltd, Japan).

### 2.5. Encapsulation efficiency determination

Encapsulation efficiency of ioversol and doxorubicin hydrochloride was calculated by measuring the amount of unencapsulated drugs as compared to the total amount added (Shen et al., 2011). Briefly, 1 mL of prepared MVLs were centrifugated at 100*g* for 10 min. The superior aqueous phase was extracted, diluted 10 times with alcohol–water (8:2), and then centrifugated at 5000 rpm for 10 min. The concentration of the supernate ioversol was quantified by HPLC analysis as the concentration of free ioversol (*C*_free_). Another 1 mL of prepared MVL suspension was directly diluted 100 times with alcohol–water (8:2), vortexed for several seconds and centrifugated before analysis. The concentration of total ioversol (*C*_total_) was quantified by HPLC analysis as well. The encapsulation efficiency of ioversol (En-iov %) was estimated by the following equation:

$$\text{En-iov}\left(\text{\%}\right)\text{ =(}C_{\text{total-iov}}\text{×10-}C_{\text{free-iov}}）\text{/}\left(C_{\text{total}}\text{×10}\right)\text{×100\%;}$$

For doxorubicin hydrochloride, the encapsulation efficiency was also determined as shown above with a little difference. MVL suspension and superior aqueous phase after centrifugation were diluted 10 times with the same multiple. The encapsulation efficiency of doxorubicin hydrochloride (En-dox%) was estimated by the following equation:

$$\text{En-dox}\left(\text{\%}\right)\text{ =}\left(C_{\text{total-dox}}–C_{\text{free-dox}}\right)\text{/}C_{\text{total-dox}}\text{×100\%}\text{.}$$

### 2.6. Determination of ioversol and doxorubicin hydrochloride by RP-HPLC

Zorbax-C18 column (15 × 0.46 cm, 5 µm particle, Agilent, USA) with a guard column was used on Agilent 1200 HPLC system. For ioversol determination, acetonitrile (A) and water (B) were used as eluted solution. The gradients were started from a 8-min plateau at 4% A, and the volume of A was increased from 4% to 100% in 5 min, following by a 3-min plateau at 100% A. 100% A was then changed back to 4% in 2 min with 10 min wash time to equilibrate the column. The flow rate was 1 mL/min and the injection volume was 20 µL. A wavelength of 254 nm was chosen for detection. The column temperature was 30 °C. For the determination of doxorubicin hydrochloride, the initial elute solvent was a mixture of 57% methanol (C) and 43% pH 4.0 acetate amine buffer (D), changed to 83% (C) and 17%(D) in 8 min, followed by a sharp variety in 0.5 min from 83% (C) to 95% (C). After 5.5-min plateau elution at 95% (C), the mixture was changed back to initial solvent mixture with a post time 10 min. The flow rate was 1 mL/min and the column temperature was 25 °C. A wavelength of 480 nm was chosen for detection.

### 2.7. In vitro drug release study

Each 5 mL prepared MVLs suspension was centrifuged at 100*g* for 10 min and washed twice with release medium to remove unencapsulated drug. Then the collected pellets were pipetted into the dialysis filters (SnakeSkin Pleated Dialysis Tubling, Thermo, USA), and dispersed with 500 mL release mediums in beakers at 37 °C, under constant rotation of 75 r/min (RC806 disolution tester, Tianjin Tianda Tianfa Technology Co., Ltd., Tianjin, China). Two different release mediums, pH 7.4 PBS buffer and pH 7.4 PBS buffer with 10% FBS containing 0.1% NaN_3_ were used in this study. Samples of an amount of 0.5 mL solution were collected at each time point (0.5, 1, 2, 4, 8, 12, 24, 48, 72, 96, 120, 144, and 168 h). The collected samples were used for determining the concentration of ioversol and doxorubicin hydrochloride by RP-HPLC as shown earlier.

### 2.8. Morphologic stability and resistance to deformation in vitro

Briefly, 100 μL aliquots of the MVLs were centrifugated under different gravity force (100, 300, 500, 1000, 3000, 5000*g*) for 15 min, transferred to dialysis tubing, and then dispersed with 50 mL of PBS (pH 7.4) release mediums in beakers at 37 °C, under constant rotation of 75 r/min (RC806 disolution tester, Tianjin Tianda Tianfa Technology Co., Ltd., Tianjin, China) for 12 h. The release behavior of encapsulated drug after centrifugation was estimated by determination as shown earlier. The morphology of collected pellets was observed using optical microscope.

### 2.9. Transcatheter embolization procedure

The embolization procedure was performed under the guide of digital subtraction angiography (Siemens AXIOM Artis FA DSA, Siemens Medical Systems, Erlangen, Germany). Rabbits were anesthetized as described earlier. Vascular access was achieved in the femoral artery through surgical cut down. Celiac angiography was performed to identify the hepatic arterial anatomy and the feeder artery of the tumor using a 3-F catheter (Cook, Bloomington, India). The left hepatic artery, which exclusively supplies blood flow to the tumor, was catheterized selectively. When the catheter was adequately positioned in the left hepatic artery after celiac arteriography was performed, MVLs or 0.9% sodium chloride was injected carefully into the artery according to different groups. Digital spot images were obtained after embolization. The catheter was then removed, and the femoral artery was ligated.

### 2.10. MR imaging

MR imaging was performed before treatment and on 4th day, 11th day after treatment by using a 3.0-T MRI scanner (Siemens 3.0 Tesla Trio Tim, Siemens Medical Systems, Erlangen, Germany) and 8-channel knee coil. Transverse spinecho T1-weighted images (repetition time/echo time, 550/8.3 ms; FOV = 200 mm × 200 mm, slice thickness =3 mm, slice gap= 0.9 mm, matrix = 320 × 192) and fast-spin-echo T2-weighted images (2000/94 ms; 200 mm × 200 mm, 3 mm, 0.9 mm, 320 × 195) were performed. The size of tumor was measured on the T2-weighted image. The tumor volume was calculated as *V* = 0.5*ab*^2^, where *a* is the longest diameter and *b* is the shortest diameter. The growth rate of the tumor was calculated using the following formula: TGR (tumor growth rate) **=***V*_-post_/*V*_-pre_ × 100%.

### 2.11. Pathological test

After completion of the last MR imaging experiments, the liver was carefully dissected, excised, and fixed in a 10% buffered formaldehyde solution. The liver was sliced at 3–4 mm intervals in the axial plane to correspond to the plane of the MR images. The liver containing tumor nodules, which were easily identified as hard, white areas, was sliced in a similar fashion and then entirely embedded for histologic examination. Sections of 5 μm thickness were stained with hematoxylin and eosin.

## 3. Results and discussion

### 3.1. The morphology and particle size distribution of IVO-DOX-MVLs

**Figure 2.**
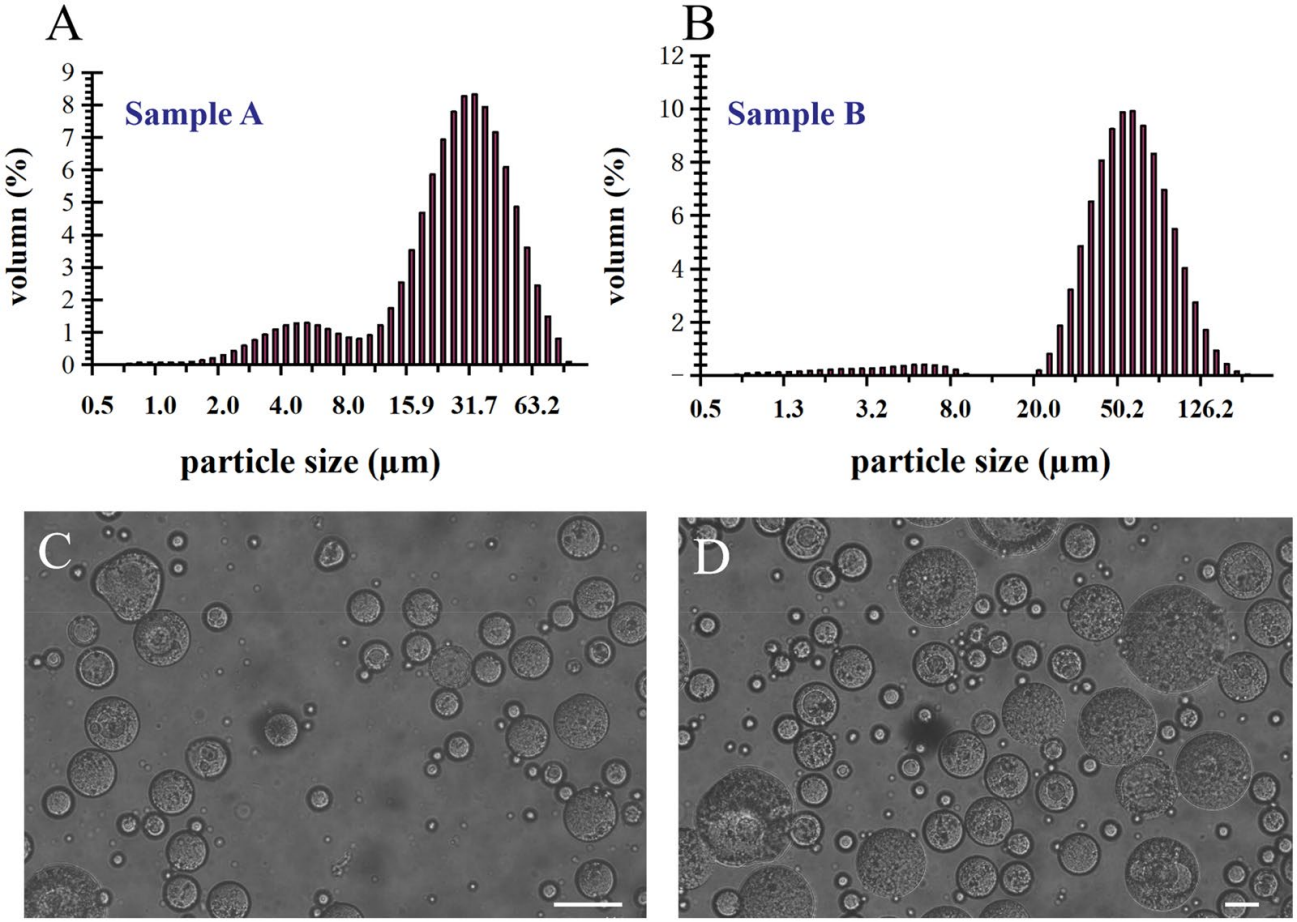
The mean particle size distribution of sample A (A) and sample B (B) determined by the Mastersizer 2000 particle size analyzer (sample A:27.0 ± 0.3 μm) and (sample B: 55.0 ± 1.1 μm). The morphology images of sample A (C) of small particle size and sample B (D) of large particle size under × 400 optical microscope (bar = 20 μm).

During w/o/w emulsion preparation, two MVLs of different sizes were obtained by changing stirring time (20–30 μm, 12 sec; 50–60 μm, 6 sec). The MVLs were spherical particles with rough appearance and the mean particle size was 27.0 ± 0.3 μm (sample A) and 55.0 ± 1.1 μm (sample B) respectively by the determination of Mastersizer 2000 particle size analyzer, as shown in Figure 2(A,B). Figure 2(C,D) is the morphology image of MVLs of different particle sizes under the ×400 optical microscope.

Here, DOPC of low phase transition temperature (–20 °C),which was the main lipid component of DepoCyte® and DepoDur® (Bulbake et al., 2017) in market, was replaced with HSPC of high phase transition temperature (55 °C). The proportion of ingredients (HSPC, cholesterol, DPPG, and triolein) was adjusted and optimized. High content of hydrophobic substance, especially cholesterol and triolein, were used to obtain more stable and solid MVLs. In this study, the lipids composition with 41 mg HSPC, 40.5 mg cholesterol, 5 mg DPPG, and 11.25 mg triolein was applied for preparation of the MVLs embolic agents.

By changing stirring time, different sizes of IVO-DOX-MVLs were prepared. During the preparation of MVLs, the selection of mixing tube and eggplant shaped bottle, mechanical stirring and manual blowing nitrogen procedure, as well as the proficiency of experimental technician, influence the yield of MVLs. Especially in the process of manual blowing nitrogen and shaking the nitrogen flask to remove chloroform, the distribution uniformity of particle size is poor, so it is necessary to use cell strainers to collect evenly large particles, excluding small particles from 1 μm to 10 μm. The adjustment of formulation and preparation parameters of MVLs was mainly referred to the article published by Sun et al. (2013). By observation of the morphology of prepared MVLs and the drug encapsulation rate, the structure of particles could be disrupted after centrifugation and the optimal formulation was finally determined. The size distribution of the obtained samples is also related to the shape of the tube and bottle chosen during preparation, as this will affect the efficiency of mechanical shear force.

The internal microstructural features of MVLs have been well analyzed. Spector et al. (1996) performed a meticulous characterization of MVLs with the composition of DOPC, DPPG, cholesterol, and triolein. Freeze-fracture TEM was employed to observe the internal closed aqueous compartments of MVLs. The shape distribution characteristics of polyhedral compartments from the number of vertices, lines, and surfaces were also analyzed. The structural features of the MVLs observed using the freeze-fracture TEM are almost consistent with the microstructural features from the computer simulation. It is also possible to label phospholipid structures and aqueous phase vesicles with lipid-soluble fluorescent dyes and water-soluble fluorescent dyes, respectively, for the observation by fluorescence microscopy to confirm that MVLs consisted of continuous phospholipid structures, and noncontinuous closed aqueous phase compartments (Mantripragada, 2002). Using the freeze-fracture TEM, the precise distribution position of triolein in the microstructure of MVLs was observed by Li et al. (2019). Triglycerides play a very important role in the structure formation of MVLs. It is used to stabilize the structure of MVLs and exists at the intersection of lipid layers that segment individual aqueous vesicles. If the content of triglycerides is too little, this will not be enough to stabilize the structure of the lipid junction of the MVLs. If the content of triglycerides is too much, and it would disrupt the lipid bilayer structure and the mobility of the cavity.

### 3.2. Encapsulation efficiency of ioversol and doxorubicin in MVLs

Specifically, linear range and precision of HPLC method of ioversol and doxorubicin hydrochloride was investigated respectively and met the requirements. Table 1 lists regression equations and encapsulation efficiency of ioversol and doxorubicin hydrochloride. The encapsulation efficiency of ioversol were 72.6 ± 5.0% in sample A and 73.7 ± 4.7% in sample B respectively. Doxorubicin hydrochloride were 99.4 ± 0.01% (sample A) and 99.5 ± 0.08% (sample B) respectively, nearly 100% encapsulation of doxorubicin hydrochloride during the preparation of IVO-DOX -MVLs. These data showed different encapsulation efficiency when different active agents were co-loaded in MVLs at the same time. Different encapsulation rates of the two drugs may due to various physicochemical properties, such as octanol–water partition coefficient, which may influence the across lipid membrane behavior (Grabarnick Portnoy et al., 2021).

**Table 1.** Regression equations and encapsulation efficiency of ioversol and doxorubicin hydrochloride in MVLs.

Drug	Linear range (μg/mL)	Regression equations (n = 5)	RSD	Encapsulation efficiency (%)	
				Sample A	Sample B
Ioversol	2.0–108	*y* = 34.802*x* + 60.541 (*R*^2^ = 0.9999)	<1.1%	72.4 ± 5.0	73.7 ± 4.6
Doxorubicin hydrochloride	0.5–20	*Y* = 119.12*x* – 37.975 (*R*^2^ = 0.9996)	<3.1%	99.6 ± 0.08	99.5 ± 0.01

**Figure 3.**
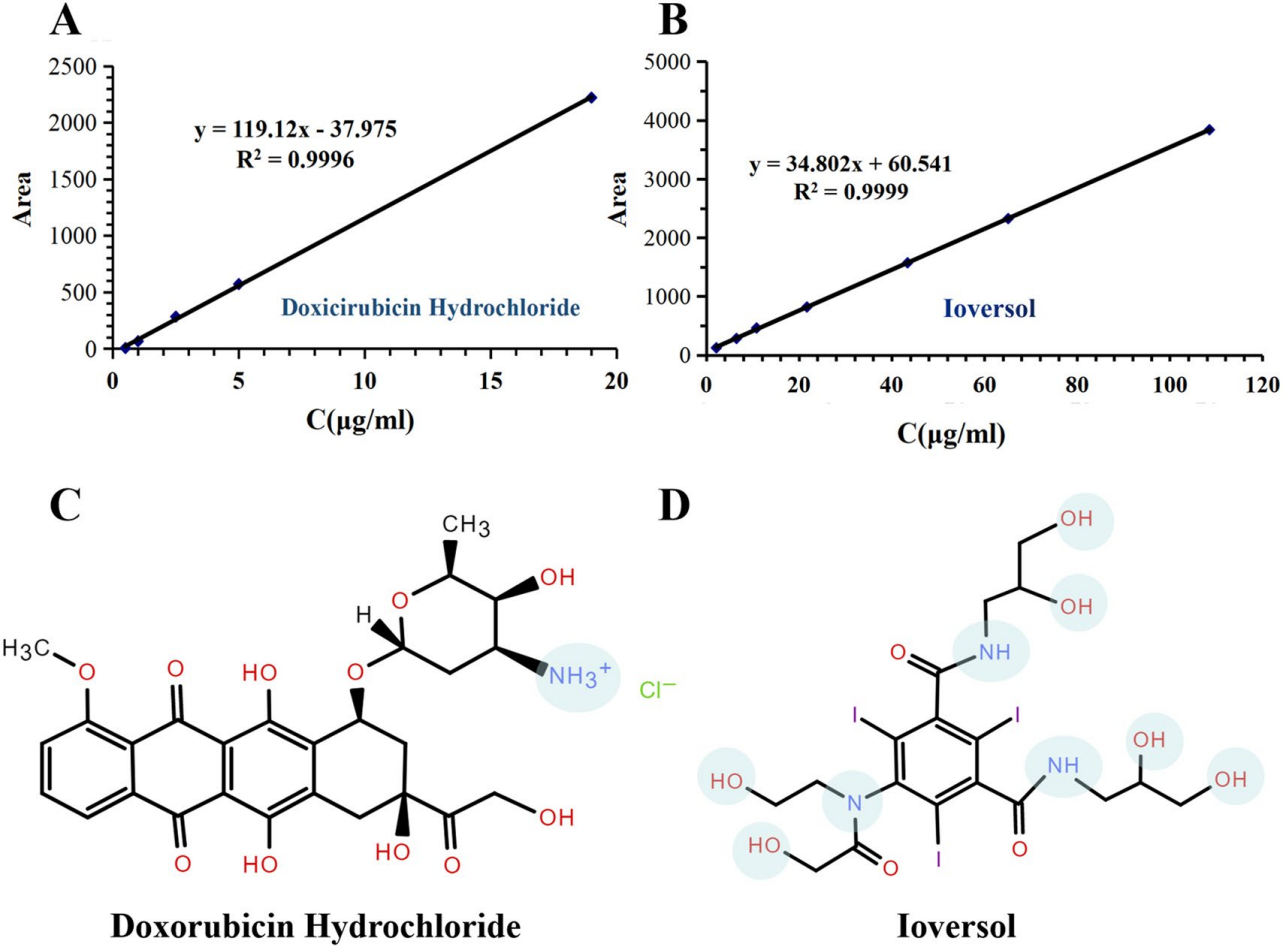
(A and B) are regression equations of doxorubicin hydrochloride and ioversol, respectively; (C and D) are the chemical structure of doxorubicin hydrochloride and ioversol, respectively. The light blue circles mark the hydroxyl and amino groups that affect *n*-octanol–water partition coefficient and dissociation constant.

As shown in Figure 3(C,D), the chemical structure formulas of doxorubicin hydrochloride and ioversol are shown respectively. Chemdraw Professional 20.0.0.41 was used to predict their chemical information. The p*K*_a_ and log*P* values of doxorubicin and ioversol were (5.085, −1.34) and (12.21, 0.21), respectively. The ioversol injection used has a pH of 6–7.4. Under this pH condition, doxorubicin hydrochloride is encapsulated in the phospholipid membrane and enclosed in the form of coexistence of ionic and free states. It is difficult to transmembrane transport during the formation of emulsion and nitrogen flux, so the encapsulation rate is high. Compared with doxorubicin hydrochloride, ioversol has a higher *n*-octanol–water partition coefficient (log*P*), that is easy to distribute into the organic phase, so the encapsulation rate is low. Doxorubicin liposomes (Wang et al., 2022) have been marketed as an active drug carrier using pH gradient. During prescription screening, the yield and structural stability of MVLs were worse with the increase of doxorubicin hydrochloride concentration, which may be related to the influence of high ionic concentration of doxorubicin hydrochloride on the stability of phospholipid structure. Claessens et al. (2004) studied the effect of different salt ion concentrations on vesicle size formed by DOPG and DOPC. The experimental results showed that the size of vesicle composed of DOPG/DOPC increased with the increase of salt concentration in the dispersion medium. The particle size of vesicles is correlated with the mean bending modulus *k*_c_ of the phospholipid bilayers. Therefore, when using phospholipid materials to encapsulate drugs, the influence of the ionizability of drugs on the size of formed vesicles should be fully considered. Especially for MVLs, it will directly affect the size of encapsulated internal aqueous vesicles, thus affecting the stability of the prescription and the bending stiffness of the phospholipid layer. Therefore, in order to improve the loading efficiency of ionic drugs, it is necessary to re-optimize the ratio of lipid components.

### 3.3. In vitro release of ioversol and doxorubicin hydrochloride from MVLs

**Figure 4.**
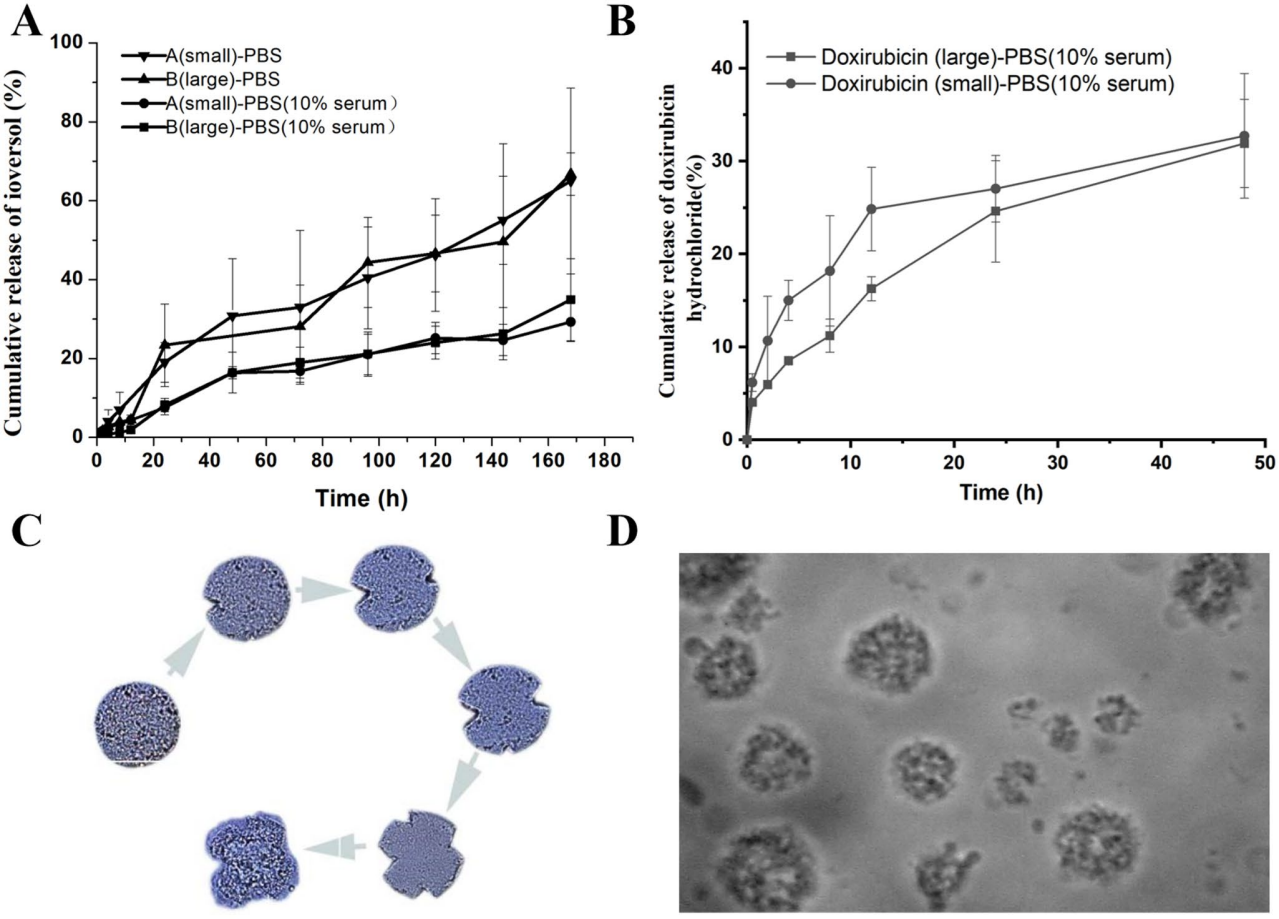
(A) In vitro release profiles of ioversol and doxorubicin hydrochloride in IVO-DOX-MVLs in PBS (pH 7.4) and PBS containing 10% serum (pH 7.4) at various time points (0, 0.5, 2, 4, 8, 12, 24, 48, 72, 96, 120,144, and 168 h); (B) Cumulative release of doxorubicin hydrochloride in MVLs within 48 h. Bars are the mean ± SD of three independent experiments; (C and D) Morphologic change of IVO-DOX-MVLs during the releasing period.

In vitro release profiles of ioversol and doxorubicin hydrochloride in PBS (pH 7.4) and PBS containing 10% blood serum (pH 7.4) are shown in Figure 4(A,B). Figure 3(A) illustrated the sustained release profile of ioversol in MVLs during 7 days. Release rate of ioversol in both sample A (small) and sample B (large) was faster in PBS (pH7.4) about 60–70% of cumulative release, when compared to that in PBS containing 10% blood serum (pH7.4), which is about 30–40% at the 7th day. These suggested the release behavior was attributed to not only particle size, but also the release medium.

The initial burst release of doxorubicin hydrochloride in MVLs within 48 h was also investigated in PBS containing 10% blood serum (pH7.4) as Figure 4(B) showed. Though doxorubicin hydrochloride can be encapsulated completely, it seems more liable to leak at initial one hour, especially in MVLs with small particle size, due to the larger specific surface area. In contrary, ioversol exhibited more steady release rate, only with a slight increase in the first hour in MVLs samples with small particle size (Figure 4A). After 24 h, the release rates of both drugs arrived at a stable level. Doxorubicin hydrochloride showed a little faster release rate than ioversol. As shown in Figure 4(A,B), the initial burst release of ioversol and doxorubicin hydrochloride in MVLs for 48 h was studied in PBS containing 10% serum (pH7.4). Although doxorubicin hydrochloride can be almost completely encapsulated, it appears to leak more easily in the first 1 h. In contrast, the release rate of ioversol was more stable, increasing only slightly in the first hour in small particle size MVLs samples. After 24 h, the release rates of both drugs reached a stable level. Doxorubicin hydrochloride is released slightly faster than ioversol. Theoretically, two drugs in the same vector should have similar release behavior. The difference in the release behavior of the two drugs may partly due to the samples taken for testing were prepared from different batches, resulting from inter-batch differences.

**Figure 5.**
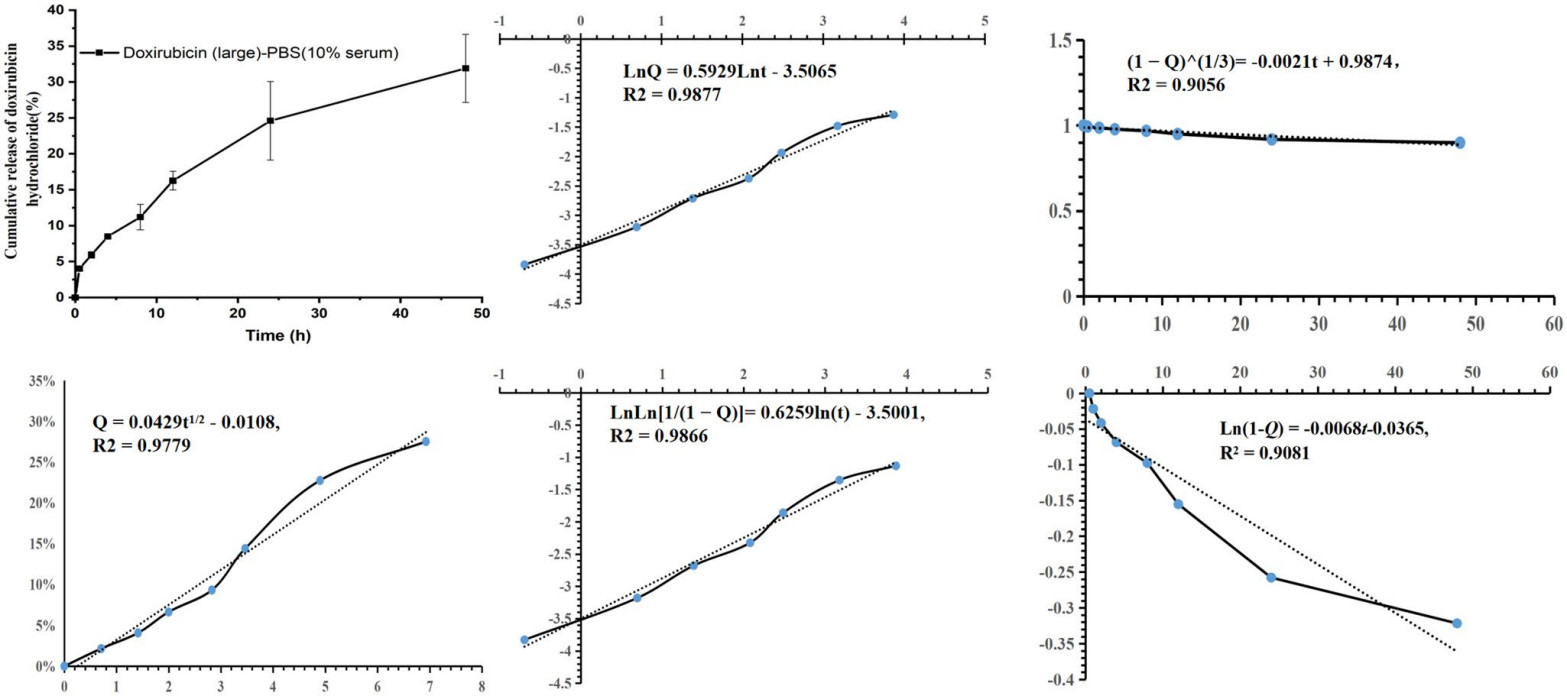
Fitting equations and correlation coefficients of cumulative release of doxorubicin hydrochloride (%) in MVLs with large particle size in PBS containing 10% FBS (pH7.4).

The cumulative release percentage of ioversol and doxorubicin hydrochloride from MVLs were fitted with different equations as shown in Table 2 and Figure 5, including the first-order kinetic equation, Higuchi equation, Weibull equation, Hixcon–Crowell equation, and Ritgar–Peppas model equation. They fitted well with Ritgar–Peppas equation, indicating that the release mechanism of the two drugs in MVLs was mainly based on the combination of erosion and diffusion. This result shows some difference from that of Sun et al. (2013) indicated. Weibull and Ritgar–Peppas equation fitted best for MVLs based on HSPC, that was significantly different from that of EPC-based ropivacaine MVLs (Shen et al., 2011). This also indicates that the choice of phospholipid excipients, lipid ratios and physiochemical properties of encapsulated drugs will significantly affect the release mechanism for MVLs.

**Table 2.** Fitting equations and correlation coefficients.

Batch no.	First order kinetics	Higuchi	Weibull	Hixcon-Crowell	Ritgar-Peppas
Large-ioversol PBS	Ln(1 – *Q*) = −0.0057*t*,*R*^2^ = 0.9494	*Q* = 0.0786*t*^1/2^ − 0.0742,*R*^2^ = 0.944	LnLn(1/(1 – *Q*)) = 0.9416Lnt– 4.9242,*R*^2^ = 0.961	(1 – *Q*)^1/3^= –0.0016*t* +0.9948,*R*^2^ = 0.9578	Ln*Q* = 0.8478Ln*t*–4.7852, *R*^2^ = 0.9603
Small-ioversol PBS	Ln(1 – *Q*) = −0.0058*t*,*R*^2^ = 0.9788	*Q* = 0.0776*t*^1/2^ – 0.0729, *R*^2^ = 0.9383	LnLn(1/(1 – *Q*)) = 1.1748Lnt– 5.7585,*R*^2^ = 0.9323	(1 – *Q*)^1/3^ = –0.0016*t* +0.9892,*R*^2^ = 0.9804	Ln*Q* = 1.1027Ln*t*–5.7097,*R*^2^ = 0.9126
Large-ioversol PBS (10% serum)	Ln(1 – *Q*) = −0.0024*t*,*R*^2^ = 0.9652	*Q* = 0.057*t*^1/2^ −0.0404,*R*^2^ = 0.9549	LnLn(1/(1 – *Q*)) = 0.9771Lnt– 5.9137,*R*^2^ = 0.9703	(1 – *Q*)^1/3^= –0.0007*t* +0.9968,*R*^2^ = 0.9652	Ln*Q* = 0.944Ln*t*–5.8846,*R*^2^ = 0.9706
Small-ioversol PBS (10% serum)	Ln(1 – *Q*) = −0.0022*t*,*R*^2^ = 0.9421	*Q* = 0.0581*t*^1/2^ −0.0549,*R*^2^ = 0.9491	LnLn(1/(1 – *Q*)) = 0.6383Lnt– 4.4194,*R*^2^ = 0.9749	(1 – *Q*)^1/3^= –0.0006*t* +0.9918,*R*^2^ = 0.9601	Ln*Q* = 0.6095Ln*t*–4.4022,*R*^2^ = 0.9776
Large-doxorubicin PBS (10% serum)	Ln(1 – *Q*) = −0.0068*t*–0.0365,*R*^2^ = 0.9081	*Q* = 0.0429*t*^1/2^ −0.0108,*R*^2^ = 0.9779	LnLn(1/(1 – *Q*)) = 0.6259Lnt– 3.5001,*R*^2^ = 0.9866	(1 – *Q*)^1/3^= –0.0021*t* +0.9874,*R*^2^ = 0.9056	Ln*Q* = 0.5929Ln*t*–3.5065,*R*^2^ = 0.9877
Small-doxorubicin PBS (10% serum)	Ln(1 – *Q*) = −0.0117*t*,*R*^2^ = 0.6129	*Q* = 0.0528*t*^1/2^ +0.037,*R*^2^ = 0.9712	LnLn(1/(1 – *Q*)) = 0.4047Lnt– 2.3009,*R*^2^ = 0.9975	(1 – *Q*)^1/3^= –0.0027*t* +0.9603,*R*^2^ = 0.7401	Ln*Q* = 0.36281Ln*t*–2.345,*R*^2^ = 0.9963

Table 3 shows the comparative analysis of Ritgar–Peppas fitting equations of IVO-DOX-MVLs and MVLs loading with other drugs. The Ritgar–Peppas fitting equation for the release of doxorubicin hydrochloride encapsulated in MVLs with small particle size has the *k* value of 0.36 (<0.45), which conforms to Fick diffusion. However, the release of ioversol encapsulated in the same prescription is different from doxorubicin hydrochloride, which reflects the combination of diffusion and erosion. Moreover, the selection of different release media, such as PBS and PBS containing 10%FBS will also significantly affect the drug release mechanism of MVLs. From Table 3, we can also observe that the release mechanism of MVLs is complex and difficult to predict. Figure 3(C) shows the morphology change of IVO-DOX-MVLs during the release of encapsulated drugs. This is obviously different from the morphology change of ropivacaine polycystic liposomes prepared by Shen et al. (2011). The *k* values of the Ritgar–Peppas fitting equations of doxorubicin hydrochloride and ropivacaine in the two prescriptions were 0.3628 and 0.7452, respectively, representing different drug release mechanisms. There are various factors affecting the release mechanism of MVLs, among which the internal structure changes and lipid recombination of MVLs should need to be studied in more detail.

**Table 3.** The Ritgar-Peppas fitting equations of drug releasing behavior of MVLs composed of diverse lipid ingredients under different release mediums.

Formulation	Drug	Particle size	Ritgar-Peppas equation	Release medium
HSPC:CHOL:DPPG:triolein (41 mg:40.5 mg:5 mg:11.25 mg)	Doxorubicin	27.0 ± 0.3 μm	Ln*Q* = 0.3628Ln*t*–2.3457, *R*^2^ = 0.9963	PBS(10% FBS)
	Ioversol	27.0 ± 0.3 μm	Ln*Q* = 0.6095Ln*t*–4.4022, *R*^2^ = 0.9776	PBS(10% FBS)
	Ioversol	27.0 ± 0.3 μm	Ln*Q* = 1.1027Ln*t*–5.7097, *R*^2^ = 0.9126	PBS
EPC:CHOL:DPPG:triolein (Sun et al., 2013) (11.7 mg:12 mg:2.3 mg:2.2 mg)	Naltrexone	12.4 µm	Ln*Q* = 0.4962Ln*t* − 2.9023, *R*^2^ = 0.9031	human plasma
	Naltrexone	12.4 µm	Ln*Q* = 0.4771 Ln*t* − 2.5153, *R*^2^ = 0.9373	saline
PC:CHOL: triolein (Shen et al., 2011)	Ropivacaine	15.36 μm	Ln*Q* = 0.7452Ln*t*–2.2837, *R*^2^ = 0.8811	PBS
DPPC:CHOL:DPPG:triolein (Wang et al., 2011) (11 mg:7.8 mg:13.9 mg:2.2 mg)	LXT-101	12.1 μm	Ln*Q* = 0.3278Ln*t*–0.8193, *R*^2^ = 0.9787	saline
DOPC:CHOL:DPPG:triolein (Qiu et al., 2005) (38 mg: 27.4 mg:5 mg:5.9 mg)	IFN α-2 b	18 µm	Ln*Q* = 0.8999Ln*t*–1.7345, *R*^2^ = 0.9828	saline
SPC:CHOL:triolein (16 mg:12 mg:4 mg) (Li et al., 2019)	Peptide	45 ∼ 55 µm	Ln*Q* = 0.4866Ln*t*–2.5293, *R*^2^ = 0.9569	saline
SPC:CHOL:triolein (16 mg:12 mg:12 mg) (Li et al., 2019)	peptide	45 ∼ 55 µm	Ln*Q* = 0.3644Ln*t*–2.2671, *R*^2^ = 0.8984	saline
SPC:CHOL:triolein (16 mg:12 mg:20 mg) (Li et al., 2019)	peptide	45 ∼ 55 µm	Ln*Q* = 0.2541Ln*t*–2.0113, *R*^2^ = 0.7442	saline

### 3.4. Morphologic stability and resistance to deformation in vitro

**Figure 6.**
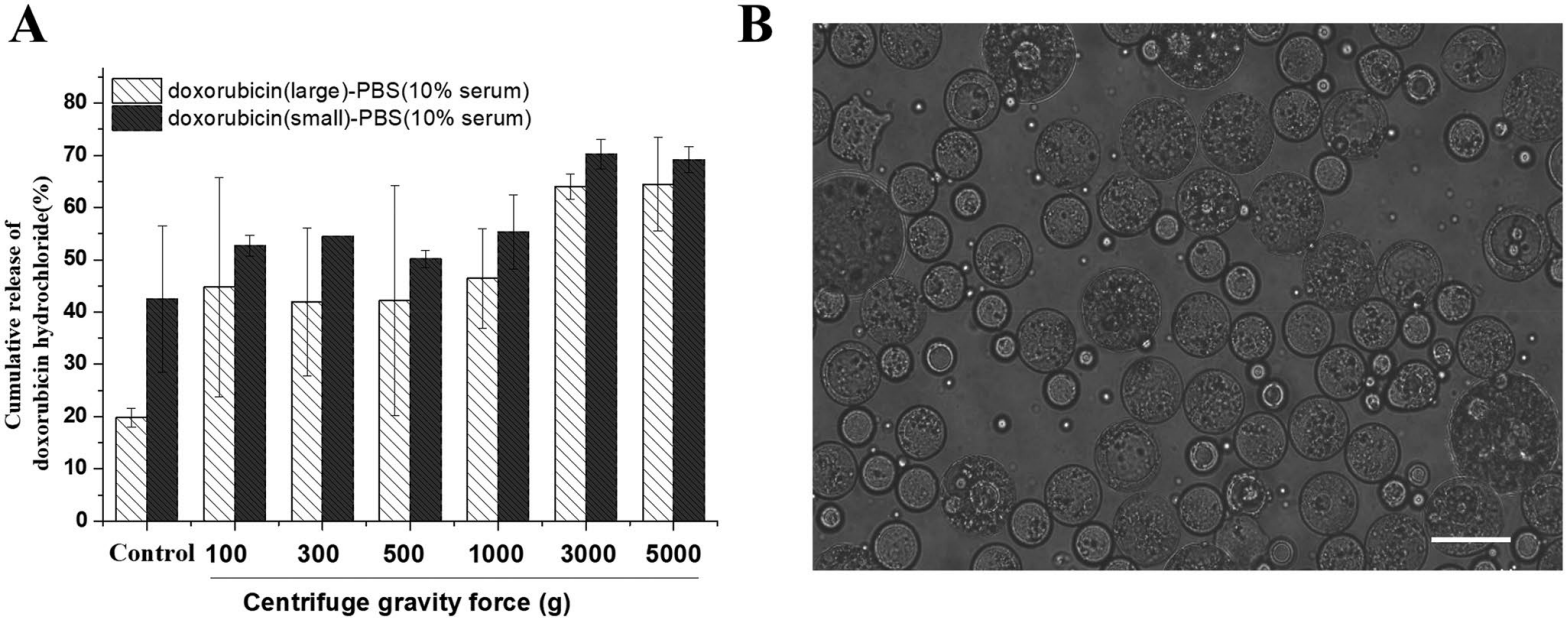
(A) Comparative study of the release behavior of doxorubicin hydrochloride in MVLs with different sizes after 15 min centrifugation at different centrifugal forces; (B) After centrifugation at 5000*g* for 15 min, the morphology of MVLs was observed under ×400 optical microscope, bar = 20 μm.

IVO-DOX-MVLs with different particle sizes showed high integrity and stability without any fragments when the morphology was observed using optical microscope after being centrifugated under different gravity force (100, 300, 500, 1000, 3000, 5000*g*) for 15 min. Figure 6(A) was the release rate of encapsulated doxorubicin hydrochloride in pH7.4 PBS solution containing 10% FBS after centrifugation under different gravity force. Both MVLs with small and large particle size showed significant increase leakage when the gravity up to 3000*g*. But there was no obvious difference between 100*g* and 1000*g* (or 3000*g* and 5000*g*). MVLs of small particles showed obviously increased leakage rate by more than 50% at 3000*g*, comparing with control group (without centrifugation). These data hinted that the inner structure of MVLs with small particle size was feasible to be destroyed under centrifugal force, which resulted in accelerated release of encapsulated drug, while the large particle ones with better elasticity and rigidity. Here, it is important to emphasize that MVLs has been subjected to multiple (≥2) centrifugations of 100*g* × 10 min during sample preparation. After centrifugation at 5000*g* for 15 min, the morphology of MVLs was observed without obvious disruption under ×400 optical microscope, as Figure 6(B) exhibits.

### 3.5. Embolization effect of MVLs in rabbit VX2 liver tumor

**Figure 7.**
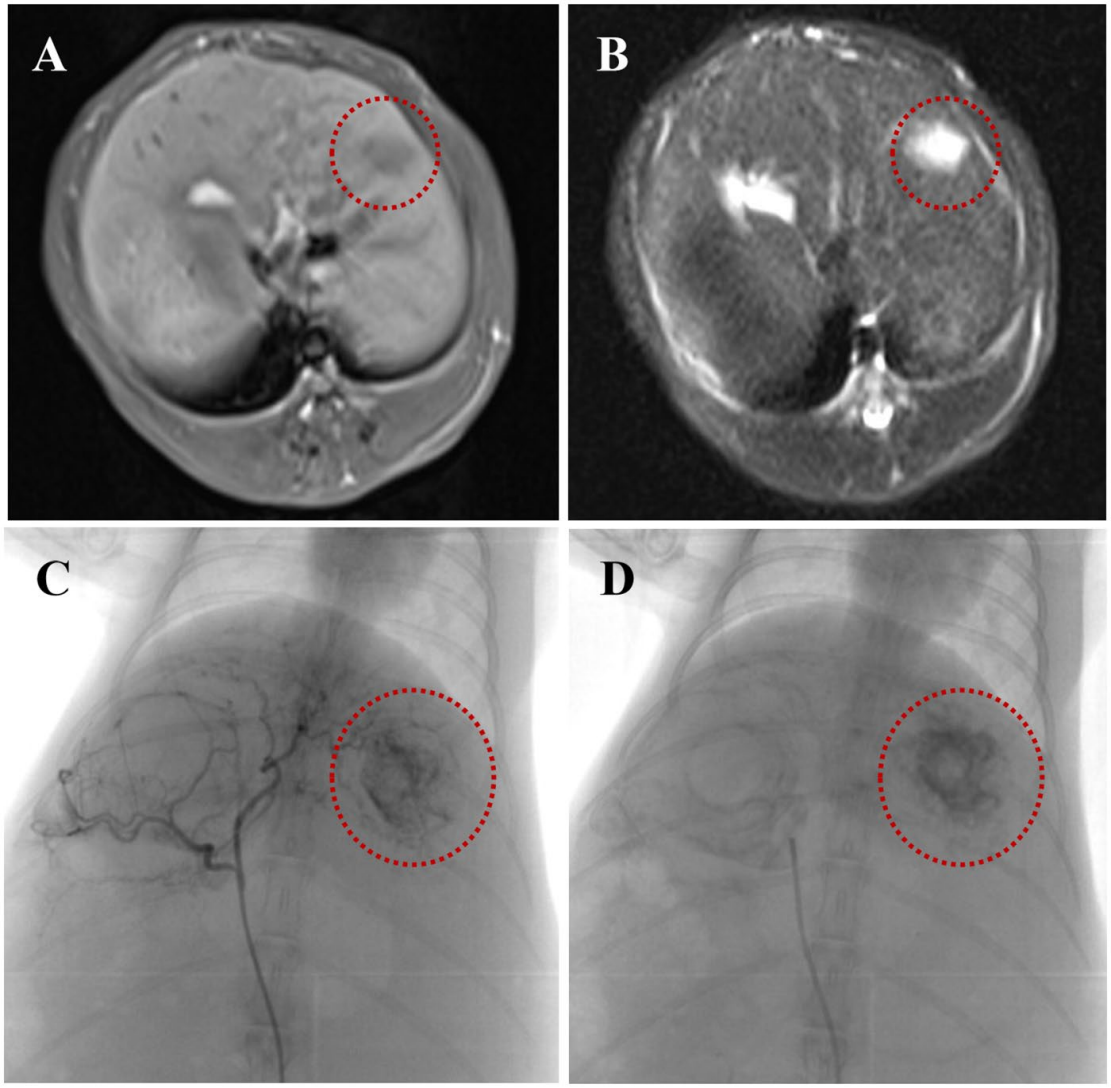
Conventional MRI images of rabbit VX2 liver tumor after implantation for 2 weeks: (A) round low-signal nodules in the left lobe of the liver on T1WI, (B) heterogeneous high-signal tumors on T2WI; (C) Hepatic arteriography showed that the tumor was supplied by the left hepatic artery with abundant blood supply and irregular contrast agent filling areas in the tumor; (D) The retention of MVLs in the tumor area observed in the retaken angiography after trans-hepatic arterial injection of MVLs.

The tumor growth status of rabbit VX2 model was evaluated by MR and CT. The tumor tissue was circular or elliptical, with hypointensity on T1WI and heterogeneous hyperintensity on T2WI (Figure 7(A,B)). In the embolization procedure, the right inguinal skin was cut open, and the right femoral artery was exposed. A 3-F SP catheter was used to super selective intubation to the left hepatic artery under DSA. A small number of experimental rabbits had vasospasm during this process, and lidocaine 10 mg was pushed through the catheter. The operation was performed successfully after the vasospasm was relieved for 5 minutes. Angiography of the hepatic artery showed abundant blood supply to the tumor (Figure 7(C)). The blood supply artery was significantly thickened and disarranged, and the irregular contrast agent filling area, namely the tumor vascular lake, was seen in the tumor. Intense staining of intra hepatic tumor after trans-arterial injection of MVLs was seen in both group A and B (Figure 7(D)), showing the retainment of MVLs in the tumor area.

**Figure 8.**
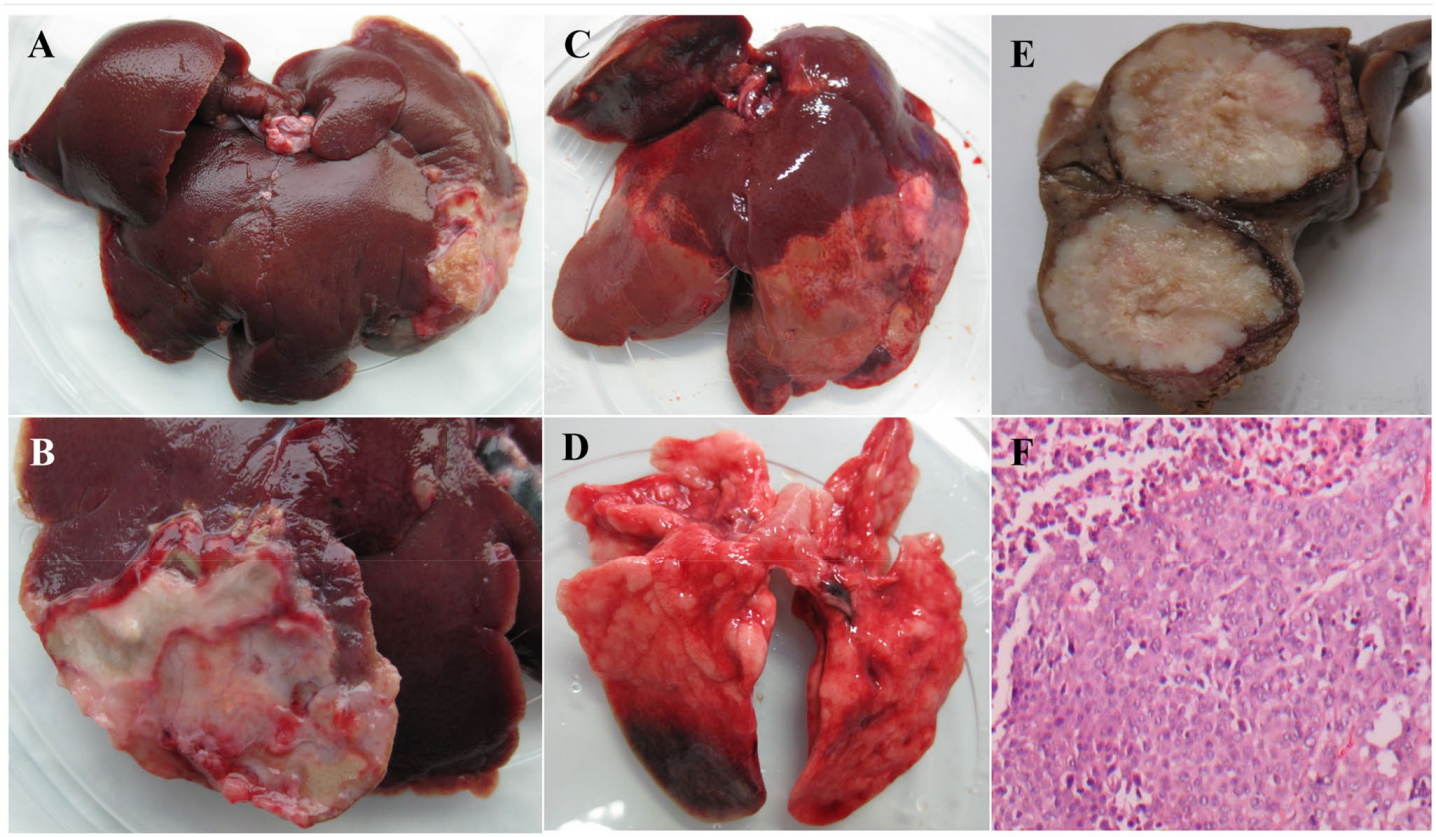
(A and B) are gross liver specimens of rabbit treated with large-size MVLs (group B), showing the extent of yellow-white infarcts (A) and the backside of liver embolism (B); (C and D) are gross liver specimens of rabbit treated with small-size MVLs (group A), showing yellow-white infarcts with large infarcts distributed at the edge of the liver lobe (C) and small embolic foci in the lungs (D).

One rabbit in group A was dead on the second day after treatment. On anatomic examination, multiple irregular-shaped infarcts and hemorrhagic focus were observed, especially at the peripheral edge of the liver lobes (Figure 8(C)). Bile duct obstruction occurred. Ulcers were found in gastric and duodenal wall. The right lung was also affected with micro infarcts in the inferior lobe (Figure 8(D)). The other rabbits were sacrificed on the 11th day after treatment. The incidence of nontarget embolization is shown in Table 4.

**Table 4.** Site of Nontarget Embolization of MVLs after TACE.

Group	Size (μm)	Site of ectopic embolization							
		Nontumorous liver tissue		Lung		Stomach and duodenum		Bile duct	
A (*n* = 5)	21.74	4	80%	3	60%	4	80%	1	20%
B (*n* = 5)	54.33	1	20%	0	–	2	40%	0	–

Although there is no obvious evidence to prove that the embolization effect of MVLs with large particle size (50–60 μm) is non-inferior to small particle size (20–30 μm) of MVLs. However, due to the influence of injection velocity and tumor blood artery selection, MVLs with large particle size has less reflux and less ectopic embolization during TACE (Table 4). In one case, complete hepatic artery embolization was observed (Figure 8(A,B)) in group B. Some clinical retrospective analysis on the safety and hepatotoxicity of small embolic agents were investigated in clinical practice. According to the clinical trial data, the smaller the size of embolic agent can penetrate the capillary network, which will reduce the metastasis of liver cancer and the rate of neovascularization caused by ischemia (Chang et al., 2021).

**Figure 9.**
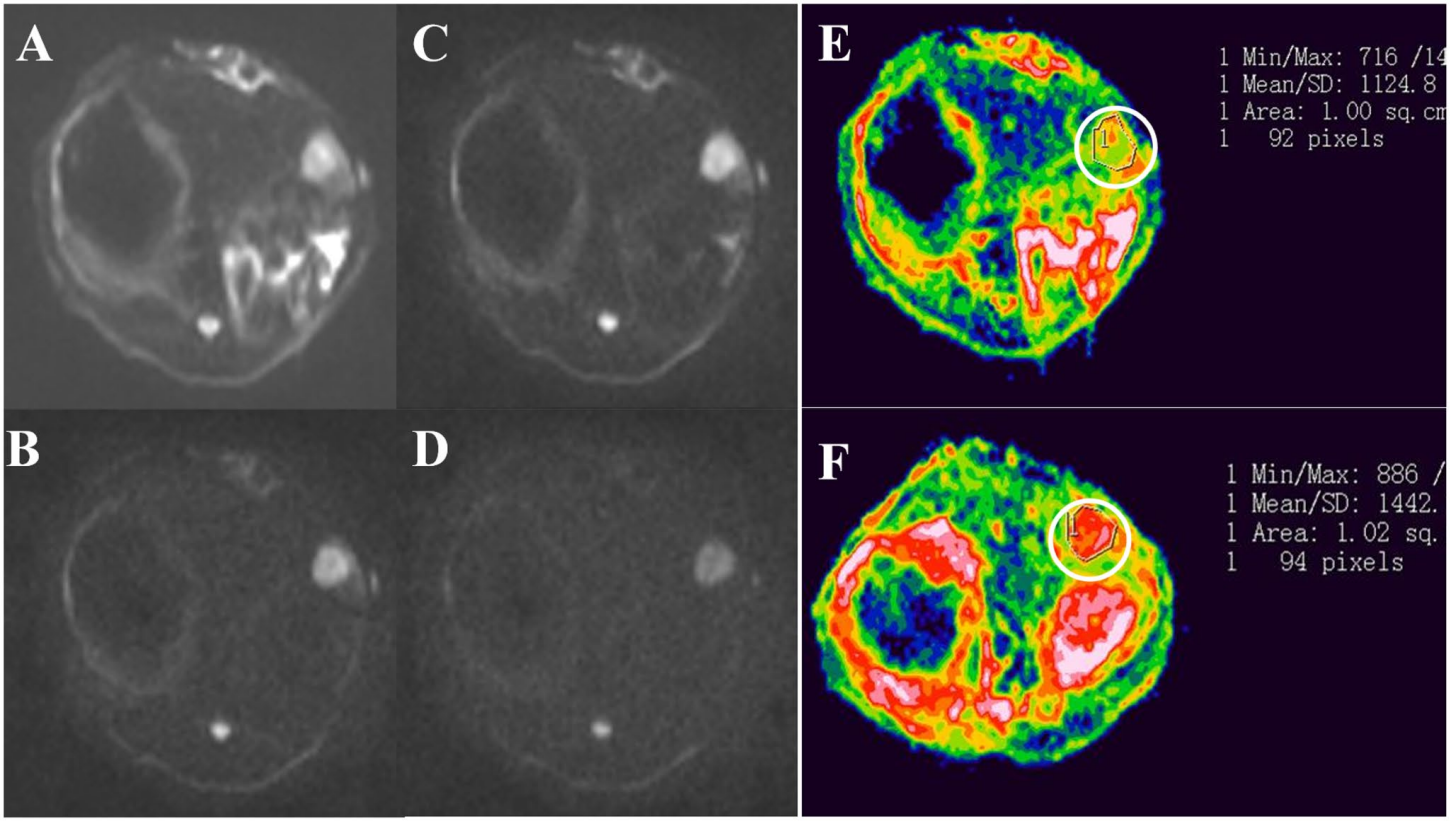
(A–D) are DWI images of the same layer of rabbit VX2 liver tumor when *b* value is 100, 600, 1000 and 1500 s/mm^2^, respectively. When *b* = 100 s/mm^2^, the normal liver parenchyma and tumor nodules were clearly distinguishable (A). With the increase of *b* value, contrast between tumor nodules and surrounding structures was obvious, but the image quality decreased, the background noise increased, and the image signal-to-noise ratio decreased (D); (E and F) are pseudo-color ADC images of experimental rabbits in MVLs group before and after the intervention, when *b* value was 1000 s/mm^2^, respectively, and the ADC value of tumors increased after the operation.

The tumor parenchyma showed high signal on DWI, and the lesions could be clearly displayed on DWI images with low *b* value and high *b* value. With the increase of *b* value, the contrast between tumor tissue and surrounding normal tissue increased, but the background noise also increased accordingly. Therefore, *b* value of 1000 s/mm^2^ was selected for analysis ADC values (Figure 9).

### 3.6. Therapeutic effect of MVLs in rabbit VX2 liver tumor

Table 5 shows the change of ADC value before and after intervention when *b* value is 1000. There is no significant difference in ADC value of VX2 tumor between each group before operation. The ADC values of group A and group B after operation is significantly higher than that before operation (*p* < .05), while the ADC value of group C has little change before and after operation (*p* > .05). There was no significant difference in ADC values between group A and B (*p* > .05). When *b* value was 1000 s/mm^2^, the correlation coefficients of ADC value and tumor necrosis rate in each group were 0.83, 0.81, and 0.82, respectively. *t* test showed that there was a linear correlation between ADC value and necrosis rate after intervention.

**Table 5.** Changes of serum ALT, AST, tumor volume, necrosis rate, and ADC values before and after intervention in each group.

Group	ALT(U/L)		AST(U/L)		ADC(mm^2^/s)		*p* value	Necrosis rate (%)	*r*	Tumor volume (mm^3^)		Growth rate (%)
	before	Day4	before	Day4	before	Day4				before	Day4	
Group A (20–30 μm)	40.36 ± 3.62	135.63 ± 8.55	20.50 ± 2.83	86.38 ± 6.28	1.05 ± 0.30	1.43 ± 0.46	.007	77.81 ± 11.22	0.83	569.24 ± 142.39	766.84 ± 225.84	135.66 ± 21.97
Group B (50–60 μm)	40.50 ± 3.67	125.12 ± 8.97	20.50 ± 3.96	78.00 ± 5.73	1.01 ± 0.16	1.41 ± 0.27	.009	80.63 ± 8.45	0.81	561.72 ± 178.82	731.82 ± 242.01	131.10 ± 17.44
Control	40.50 ± 4.31	50.86 ± 5.44	21.25 ± 2.05	27.00 ± 1.31	1.09 ± 0.26	1.01 ± 0.35	.624	32.81 ± 7.84	0.82	574.56 ± 193.86	1503.95 ± 315.77	287.47 ± 113.26

The changes of serum AST and ALT before and after treatment was no significant different in each group before operation. But at 4 days after operation, AST and ALT in each group were higher than those before treatment. MVLs group was significantly higher than normal saline group and the difference between group A and group B was statistically significant (*p* < .05).

There was no significant difference in the tumor volume of each group before operation as shown in Table 5. The tumor volumes of group A, B, and C were 766.84 ± 225.84 mm^3^, 731.82 ± 242.01 mm^3^, and 1053.95 ± 315.77mm^3^, respectively. The growth rates were 135.66 ± 21.97%, 131.10 ± 17.44%, and 287.47 ± 113.26%, respectively. The tumor volume and growth rate of group A and B were lower than those of group C, and the differences were significant (*p* < .05). There was no significant difference in tumor volume and tumor growth rate between group A and group B (*p* > .05).

**Figure 10.**
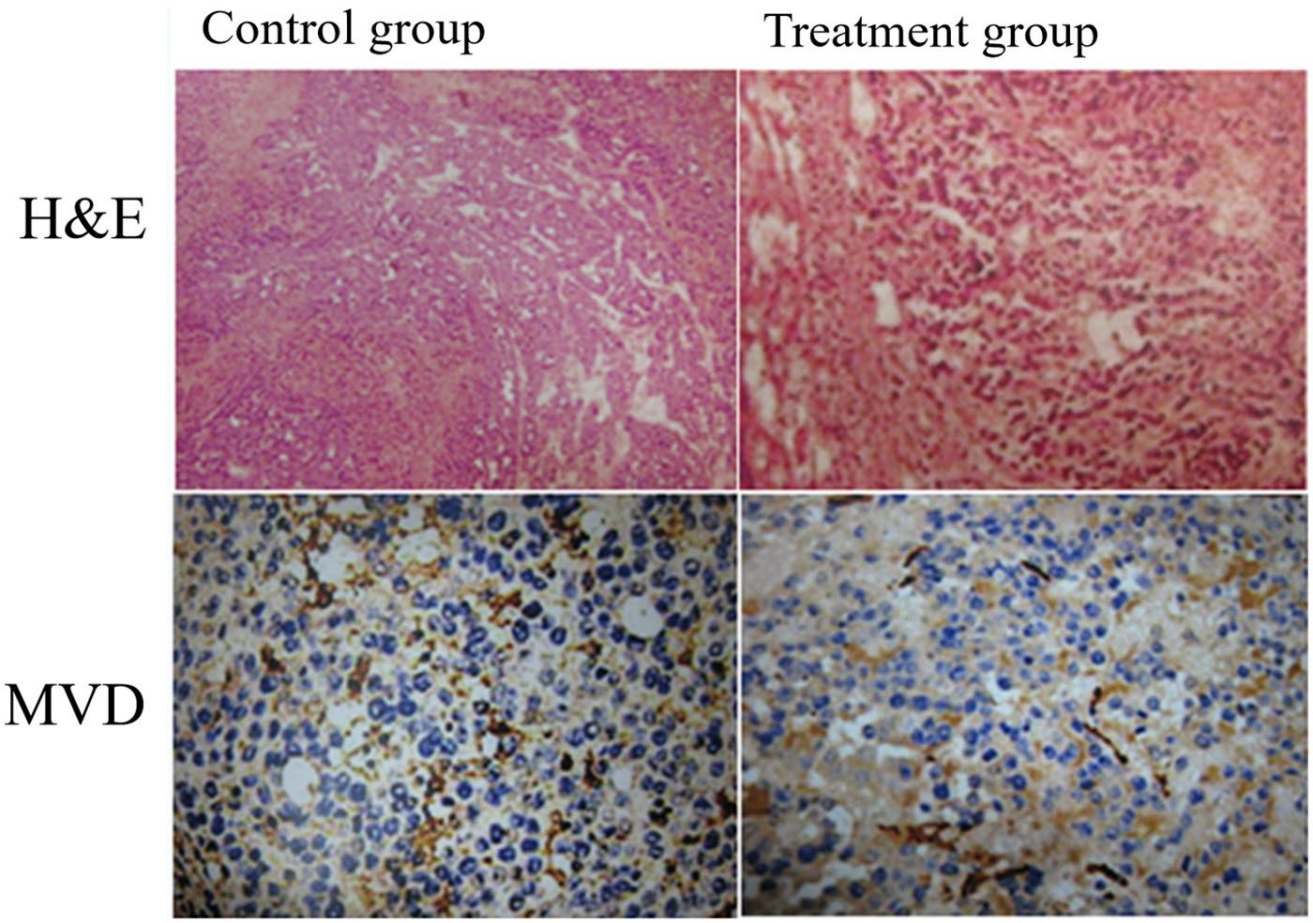
Observation of the necrosis degree and microvessel density on rabbit VX2 liver tumor model.

Large-size MVLs encapsulated with doxorubicin showed some interesting change in tumor growth in some cases. It is clear that since 1 week after TACE, the tumor volume kept decreasing significantly. H&E stained was done to estimate the necrosis degree. MVD was determined by counting the number of the microvessels. Compared with the control group, MVD value decreased obviously (Figure 10).

### 3.7. Clinical feasibility evaluation of MVLs as a novel embolic agent

TACE is the most widely used local–regional liver-directed therapy for the patient with advanced liver disease, inadequeate hepatic reserve, and medical comorbidities to improve survival rates as first-line palliative treatment (Liu et al., 2015). It involves the injection of particles or fluids that are released into the tumor-feeding artery via an intraarterially inserted catheter to occlude the target vessel, resulting in devascularization, HCC cell apoptosis, and ischemic necrosis during hypoxia (Hu et al., 2019). One of the two main categories of embolization agents widely used in clinic was solid particles, which divided into non-spherical or microspherical ones (Hu et al., 2019). The former ones usually have to cut into small pieces by manual to prepare various sizes when used for larger vascular embolization (Maeda et al., 2013). The microspherical embolic agents with uniform shape and size included poly(lactic-*co*-glycolic acid) (PLGA) (Liang et al., 2017), polyvinyl alcohol (PVA) (Chang et al., 2021), degradable starch microspheres (Yildiz et al., 2022), gelatin sponge, and tris-acryl gelatin microspheres (TAGM) (Liu et al., 2015). These microspheres can combine with anticancer drug which can be released in a controlled manner. The other category of embolization agent was the fluid one, including glues (cyanoacrylate) (Loffroy et al., 2021), gels (alginate and CaCl_2_) (Zeng et al., 2018), sclerosing agents (ethanol, acetic acid) (Sun et al., 2022), and viscous emulsions (Lipodol, Ethibloc) (Loffroy et al., 2009). But the conventional TACE embolic agents, such as oily contrast material (Lipdol), polyvinyl alcohol or gelatin sponge may induce a significant neoangiogenetic reaction, cause revascularization and metastasis because of hypoxia and incomplete embolization. Shim et al. (2008), investigated 147 patients with unresectable HCC patient prognosis to find the association between a change level of VEGF after TACE and HCC patient prognosis. Many undegradable microspheres such as PVA can increase the risk of abscess because of the persistent ischemic liver injury and inflammatory reaction of vessel embolic site due to the poor biocompatibility (Del Poggio et al., 2007). These microspheres always can’t be developed under X-ray, so more conservative judgment on the end of embolic agent should be made when carrying out TACE. When anticancer agents were added to aid the cancer killing effect, the drugs release and distribution profile from most preparations were not carefully designed. The chemotherapeutic drug was easy to wash out, contact short time with the tumor and had systemic toxicity (Chang et al., 2021). Compared to large particle size, small PVA (50 μm) or spherical embolic particles (40–120 μm) which can cause terminal vessel blockade are an effective treatment method for patients with unresectable HCC, when Maluccio et al. (2008) reviewed 322 patient with HCC underwent 766 embolizations. So a novel targeted embolizing agent used in TACE should have the following characterizes: (1) target cancer effectively or embolize tumor(s) as selectively as possible; (2) promote drug absorption; (3) control and sustain drug release;(4) increase exposure time of tumor to chemotherapy; (5) reduce systemic drug levels and subsequent undesirable side-effects and toxicity to normal tissue.

Multivesicular liposome (MVL) is a novel liposome that differs from traditional liposomes. It has unique structure of multiple non-concentric aqueous chambers surrounded by a network of lipid membranes, and is biodegradable, biocompatibility, and allows encapsulating an array of drugs or active ingredients. Many experiments have underscored the sustained, steady, and reliable release of active agents from multivesicular liposomes (Wang et al., 2011).

In this study, we showed that MVLs could also be used as an embolic agent. We replaced DOPC of low phase transition temperature (–20 °C), which was the main lipid component of DepoCyte® and Exparel® in market, with HSPC of high phase transition temperature (55 °C). Then we tested different formulations by adjusting the proportion of ingredients (HSPC, cholesterol, DPPG, and triolein), and finally got the stable formulations to prepare solid MVLs. To obtain stable structure of large particles of MVLs, more lipids are needed, especially for cholesterol and triolein.

The morphology images of MVLs with the optimized formulation were fairly spherical in shape and the surface was of a characteristic roughness as Figure 2 shows. In some testing formulations, the particle surface was smooth with less stability after centrifugation under 100*g* for 10 min or becoming disintegration during the storage period at 4 °C for several days. We obtained different sizes of MVLs by changing stirring time, and the uniformity of size distribution was not good enough containing small particles range from 1 μm to 10 μm during preparation and can be improved through cell strainers to collect more evenly large particles. MVLs with large particle size have higher uniformity than that of small ones, which may contribute to lower non-targeted embolization.

MVLs showed more stable and better sustained release efficiency under the serum condition. The morphologic stability and resistance to deformation in in vitro study showed high integrity and stability without any fragments after centrifugation at various gravity force. The encapsulation efficiency of the two drugs was also very high, especially for doxorubicin nearly to 100%. The optimized formulation of MVLs in in vitro study showed the potential ability to be a novel embolic agent.

Traditional doxorubicin embolic agent for TACE was just simple mixing, such as suspending doxorubicin in Lipdol to form w/o or w/o/w preparations. Though HCO-60 was used as emulsifier to prolong the sustained release of doxorubicin, 50% of doxorubicin was still released after 72 h compared to 100% released from w/o without HCO-60 after 24 h (Lin et al., 1992). However, doxorubicin encapsulated in MVLs displayed less than 35% cumulative release rate after 48 h, showing better sustained release performance in PBS (pH 7.4) with 10% FBS serum.

Doxorubicin was loaded together at low concentration but persistently aimed to achieve antiangiogenesis effect. Kerbel and Kamen (2004) and Shaked et al. (2005) worked on an approach that considered to deal with antiangiogenesis effect, involving administering chemotherapy drugs ‘metronomically’ below the MTD, which maintained sustained release for extended time with no breaks to have anti-angiogenic properties, e.g., celecoxib or targeted anti-VEGF anti-bodies as well as some other combinations. Park et al. (2021) investigated low-dose doxorubicin (DOX)-mediated dysregulation of endothelial progenitor cells (EPC) functions. These data suggest that regulated in development and DNA damage response 1 (REDD1) is a potential therapeutic target for the inhibition of tumor angiogenesis and responsible for the translational repression of VEGFR-2 transcript.

The animal experiment showed differences in tumor growth rate and necrotic rate between MVL groups and control group, illustrating the embolic effect of MVLs. The incidence rate of non-target embolization is lower in large particle size MVLs group. The embolization in gastric and doudenal wall may be partially due to the backflow after injection, taking no account of the complexity and twists of blood supply system of rabbit VX2 tumor model. Jang et al. (2009) also reported a case of acute ischemic duodenal ulcer when using doxorubicin and lipodiol emulsion. Shimohira et al. (2011) proposed that super-selective TACE can maximize the impact of treatment on the tumor while minimizing damage to tumor-free liver parenchyma and is preferable to nonselective therapy. Using the triaxial microcatheter method can make the level of embolization more specific. And we observed some changes of microvessel density at tumor site from pathological examination (Figure 10).

Since it is benefit to achieve the distal embolization of tumor vessels for small particles to avoid ischemic embolization more effectively. MVLs that can simultaneously encapsulate contrast agents and multiple anticancer drugs are a good choice as a new embolic agent. The marketed MVLs products will also provide experiences for the commercial production.

## 4. Conclusion

Patient prognosis with liver cancer depends not only on the tumor stage but also hepatic function reserve. To achieve greater specificity of the embolization can protect hepatic function at maximum degree, avoid hepatic artery stenosis, occlusion, and aneurysmal change. We showed that MVLs could be made with different size distributions while retaining structure stability and sustained drug release profiles. They were used as the embolic agents to treat rabbit VX2 liver tumor models. Imaging studies showed that they were well retained inside tumor vessels and resulted in tumor vessel occlusion and tissue necrosis. Though smaller size MVLs apparently had more serious side effects with many off-target embolization in non-tumorous tissues, this might be avoided by using microcatheter. In future work, we will continue to pay attention to the development of TACE as a minimally invasive interventional therapy, and use it as a new drug administration method to effectively deliver MVLs to tumor tissues directly, and evaluate the effect of metronome chemotherapy on tumor treatment and anti-neovascularization.

## Abbreviations

HCC: hepatocellular carcinoma
TACE: transcatheter arterial chemoembolization
MVLs: multivesicular liposomes
IVO-DOX-MVLs: multivesicular liposomes containing ioversol and doxorubicin hydrochloride
HPLC: high performance liquid chromatography
MR: magnetic resonance
DSA: digital subtraction angiography.
